# Effects of animal-assisted therapy on social behaviour in patients with acquired brain injury: a randomised controlled trial

**DOI:** 10.1038/s41598-019-42280-0

**Published:** 2019-04-09

**Authors:** Karin Hediger, Stefan Thommen, Cora Wagner, Jens Gaab, Margret Hund-Georgiadis

**Affiliations:** 1 Department of Psychology, University of Basel, Basel, Switzerland; 2 REHAB Basel, Clinic for neurorehabilitation and paraplegiology, Basel, Switzerland; 3 Department of Epidemiology and Public Health, Swiss Tropical and Public Health Institute, Basel, Switzerland

## Abstract

Animal-assisted therapy (AAT) is increasingly used to address impaired social competence in patients with acquired brain injury. However, the efficacy of AAT has not been tested in these patients. We used a randomised, controlled within subject trial to determine the effects of AAT on social competence in patients undergoing stationary neurorehabilitation. Participants received both AAT sessions and paralleled conventional therapy sessions. The patients’ social behaviour was systematically coded on the basis of video recordings of therapy sessions. Moreover, mood, treatment motivation and satisfaction was measured during each therapy session. We analysed 222 AAT and 219 control sessions of 19 patients with linear mixed models. Patients showed a significantly higher amount of social behaviour during AAT. Furthermore, patients’ positive emotions, verbal and non-verbal communication, mood, treatment motivation and satisfaction were increased in the presence of an animal. Neutral emotions were reduced but no effect was found regarding negative emotions. Our results show that AAT increases aspects of social competence and leads to higher emotional involvement of patients with acquired brain injury, reflected in higher social engagement, motivation and satisfaction during a therapeutic session.

## Introduction

Acquired brain injury of traumatic or non-traumatic origins is a globally significant public health issue. It is estimated that traumatic brain injury annually affects approximately 50–60 million people worldwide with an incidence rate varying between 50–640/100′000 people per year^1,2^. Given the high number of affected people and the consequences of acquired brain injuries, effective therapy is needed to avoid long term impairments. Patients with acquired brain injury have difficulties in social competence and especially in social communication skills^3^, as they involve conversational partners in conversations less often, need more direct questions, suffer from reduced emotional empathy, affective responsivity and impaired emotional expression^4,5^. Also, depression is common after brain injury^6^. Thus, reduced social integration and an increased social isolation is a major problem for patients with acquired brain injury^7^.

Animal-assisted therapy (AAT) is an innovative new method to address psychosocial skills and socioemotional functioning in patients with various deficits. Currently, this therapy form is a rapidly increasing approach in rehabilitation and may be an effective complement to conventional therapy types in neurorehabilitation^8^. To date, AAT has been evaluated in post-stroke patients with hemiparesis^9,10^, aphasia^11^, and in patients with traumatic brain injury^12^, and severe disorders of consciousness^13^. Most of the studies focused on physical functioning in combination with hippotherapy and are of low scientific quality^14^. Systematic studies investigating effects of AAT in patients with acquired brain injury are lacking, despite increasing use in rehabilitation. Previous studies show that interacting with animals leads to more displayed social behaviours, e.g. talking, looking at faces, and physical contact, as well as more positive affect in autism spectrum disorder^15,16^, dementia and intellectual disabilities^17,18^. Taken together, these findings suggest that AAT might be effective to foster social communication and interaction skills in neurorehabilitation after acquired brain injury. Recent research shows that fostering social competence in patients with acquired brain injury is highly important to decrease risks of behavioural disorders and psychiatric morbidity and enhance quality of life for survivors^19,20^ .To accomplish this goal, the patients’ active social engagement during therapy is needed^21^. This is especially important since patients with acquired brain injury can have severe deficits regarding engagement and motivation^22^. The amount of social behaviour reflecting social engagement during a therapeutic setting is therefore an important outcome in this population. Moreover, motivation and mood are both key for increased engagement and positive outcomes in rehabilitation^23,24^ and can be reflected in patient satisfaction.

The aim of this study was to investigate the effects of AAT on social competence in patients with acquired brain injury undergoing neurorehabilitation. We addressed this by measuring the displayed social behaviour as well as the mood, treatment motivation and satisfaction of the patients.

## Results

**Figure 1:**
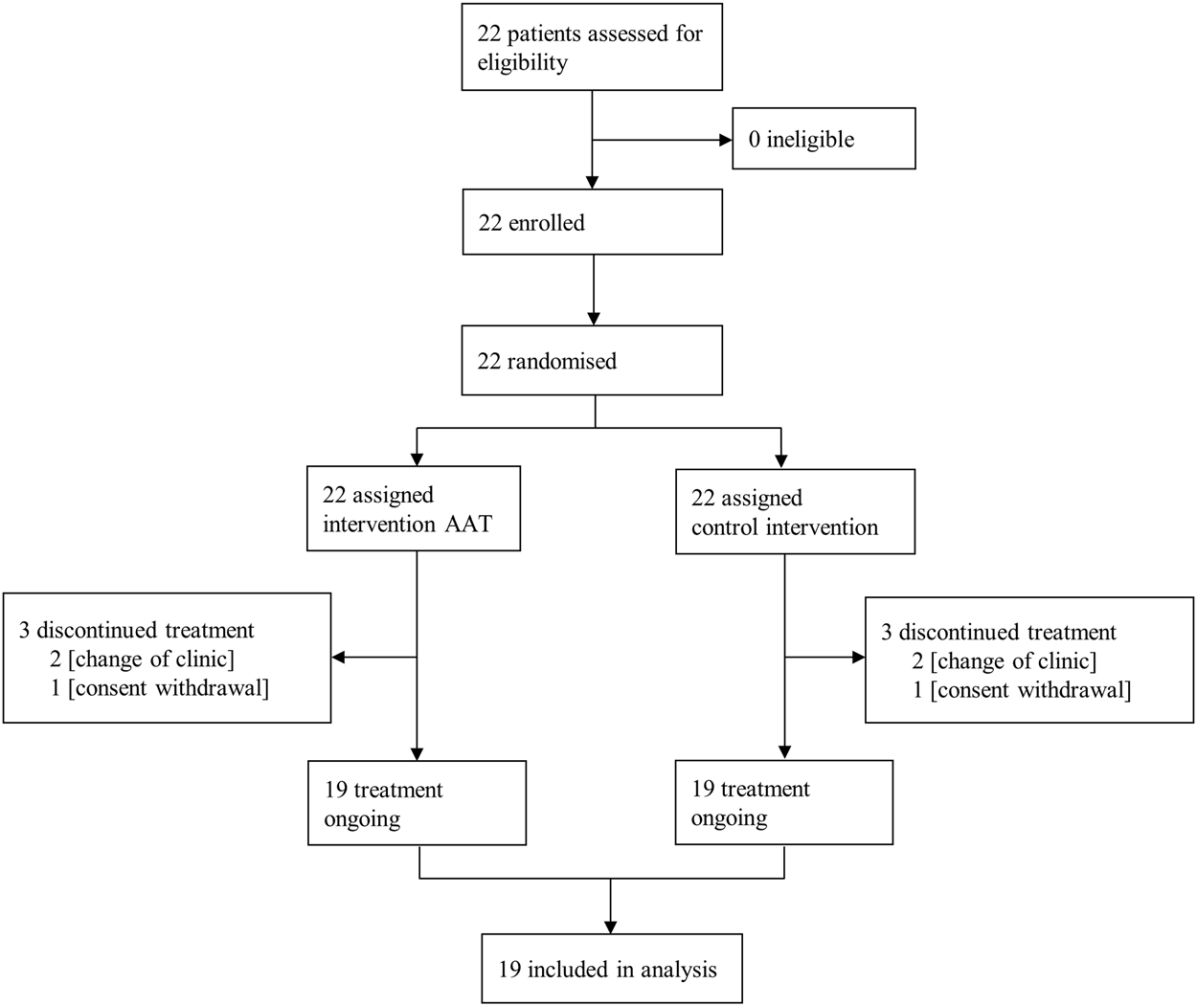
CONSORT flowchart.

Between February 24, 2014 and February 11, 2016, 22 patients were screened and randomly assigned to the study protocol. Three patients dropped out during the study (Fig. 1). The final sample of 19 patients (mean age in years (SD): 50.84 (14.40); traumatic brain injury (TBI): N = 8, non-TBI: N = 11) consisted of 13 males and 6 females (mean age in years (SD) = 52.9 (13.4); 46.5 (16.9), respectively). The Functional Independence Measure (FIM)^25^ total scores at study start ranged between 31 and 116 (M = 68.7, SD = 26.1) and FIM cognitive scores at study start ranged between 8 and 30 (M = 18.7, SD = 6.3). Patients with TBIs and non-TBIs did not differ in FIM total or FIM cognitive scores before or after the study.

**Figure 2:**
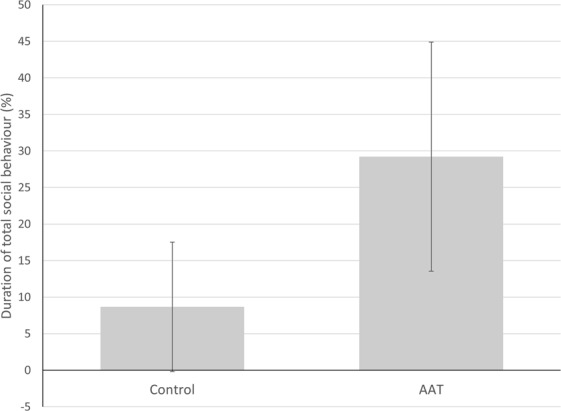
Differences in patient total social behaviour between AAT and conventional therapy sessions (control). Bars represent means with standard deviations.

**Figure 3:**
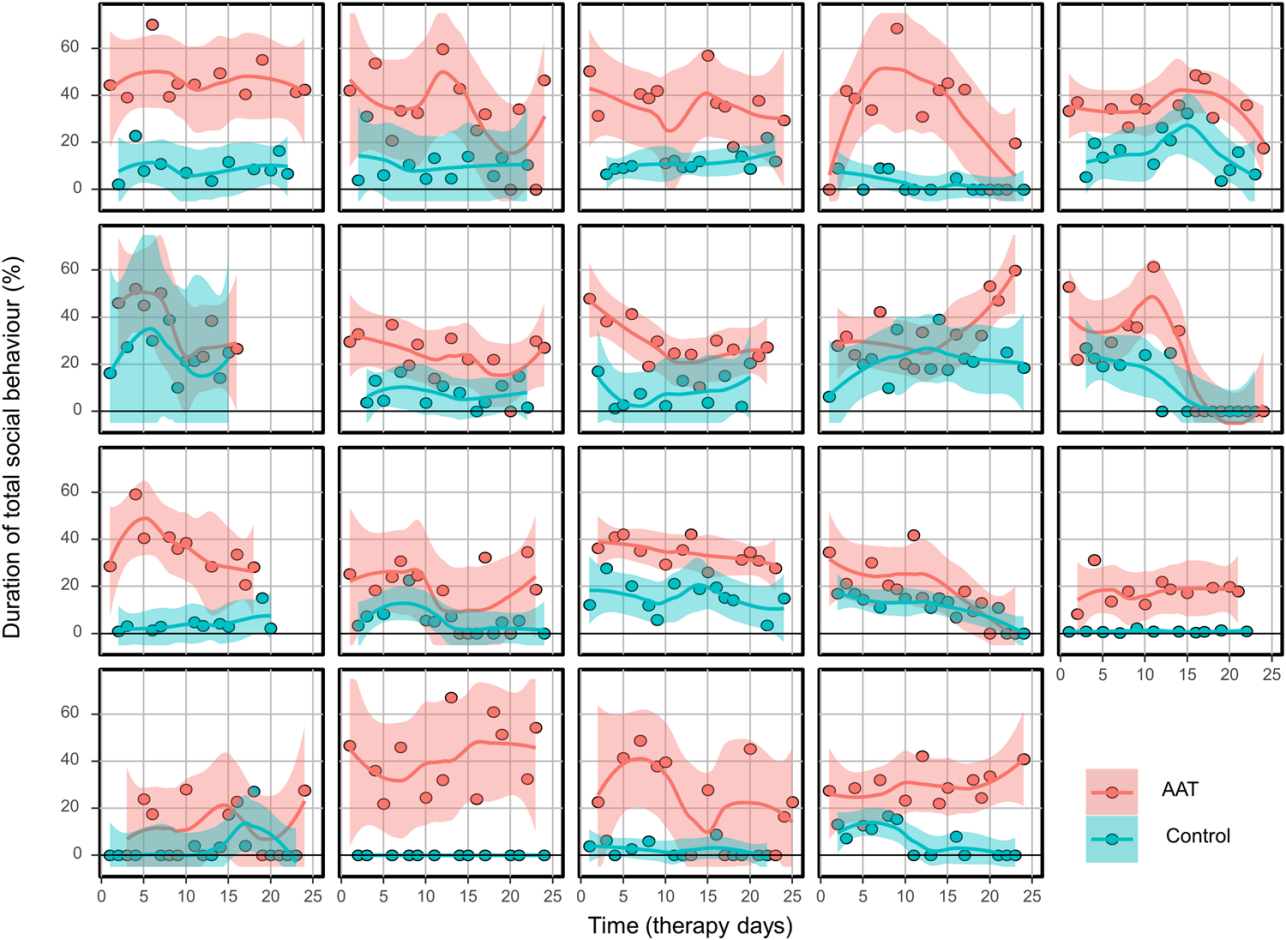
Effects of AAT and conventional therapy sessions (control) for each patient separately over the course of time. Lines and corresponding CI’s were estimated by lowess algorithm.

With regard to the primary outcome, duration of overall social behaviour was significantly higher during AAT compared to conventional therapy sessions (b = 20.47, CI = 18.32–22.62, *p* < 0.001, ICC = 0.21, Fig. 2). Mean duration of social behaviour decreased slightly over time in the AAT sessions (b = −0.46, CI = −0.67–0.24, *p* < 0.001 ICC = 0.21) but not in the conventional therapy sessions (b = −0.18, CI = −0.40–0.04, *p* = 0.109 ICC = 0.21) with a remaining large effect for condition (b = 24.04, CI = 19.78–28.30, *p* = 0.001 ICC = 0.21). Figure 3 shows the effects of AAT and conventional therapy for each patient separately.

With regard to the secondary outcomes, patient total verbal communication was significantly higher (b = 6.32, CI = 3.77–8.86, *p* < 0.001, ICC = 0.57) and active verbal communication was increased (b = 7.30, CI = 5.87–8.73, *p* < 0.001, ICC = 0.44), while reactive verbal communication was decreased (b = −2.44, CI = −3.91–−0.98, *p* = 0.001, ICC = 0.67) during AAT compared to conventional therapy sessions . Regarding non-verbal behaviour, the analysis revealed a significantly higher proportion of total non-verbal communication and interaction during AAT compared to conventional therapy sessions (b = 26.65, CI = 24.29–29.01, *p* < 0.001, ICC = 0.12). All three non-verbal behaviour categories were significantly higher during AAT (gaze: b = 42.42, CI = 39.26–45.58, *p* < 0.001, ICC = 0.08; body movement: b = 24.06, CI = 21.36–26.77, *p* < 0.001, ICC = 0.18; physical contact: b = 16.84, CI = 14.19–19.49, *p* < 0.001, ICC = 0.09). The duration of the displayed positive emotions was significantly higher with nearly doubled duration during AAT in comparison to conventional therapy sessions (b = 3.24, CI = 2.28–4.19, *p* < 0.001, ICC = 0.36), while the duration of the neutral emotions was reduced (b = −2.97, CI = −3.97–−1.97, *p* < 0.001, ICC = 0.34). There was no effect regarding negative emotions.

The MDBF mood scale showed that patients had significantly better mood during AAT compared to conventional therapy sessions (control: M(SD) = 16.12 (4.29); AAT: M(SD) = 16.93 (4.20); b = 0.84, CI = 0.41–1.27, *p* < 0.001, ICC = 0.74). Patients also rated themselves as significantly more motivated (control: M(SD) = 132.22 (28.21); AAT: M(SD) = 138.80 (27.13); b = 6.59, CI = 3.70–9.47, *p* < 0.001, ICC = 0.70) and significantly more satisfied (control: M(SD) = 124.54 (37.81); AAT: M(SD) = 129.81 (23.80); b = 5.18, CI = 1.55–8.82, *p* = 0.005, ICC = 0.77). The assessment of the therapists regarding patient’s treatment motivation (control: M(SD) = 234.56 (28.21); AAT: M(SD) = 141.83 (19.95); b = 7.24, CI = 3.84–10.64, *p* < 0.001, ICC = 0.34) and satisfaction (control: M(SD) = 128.14 (27.75); AAT: M(SD) = 137.31 (23.80); b = 9.35, CI = 5.60–13.10, *p* < 0.001, ICC = 0.42) revealed the same results.

## Discussion

Integrating animals into therapy sessions for patients with acquired brain injury led to significantly more social behaviour during AAT, with an increase in both verbal and non-verbal communication. Moreover, patients displayed significantly more positive emotions, were more motivated and rated their satisfaction level higher during AAT.

Our findings are in line with previous reports in different clinical populations^15–18^ and an earlier review on AAT in neurorehabilitation, which documents improvements in social functioning and interaction in patients suffering from cerebral palsy, pervasive developmental disorders, multiple sclerosis, spinal cord injury, stroke, and mental disorders^8^.

The observed beneficial effects of AAT are possible due to different processes. First, animals can become relevant therapeutic partners for patients, who are often highly motivated to care for the animal. Interestingly, this effect is especially seen in populations that are difficult to reach with standard therapies, either because they have problems with social interactions or because they lack cognitive functions and/or verbal communication^15–18,26^. Since animals communicate non-verbally and are non-evaluative, they are especially suitable for patients with acquired brain injury, who often have trouble connecting verbally, struggle with feelings of shame or may be highly alert to social evaluation. Second, animals provide motivation for therapeutic activities. Patients have difficulties with activities of daily life, such as getting dressed, cooking or eating, but are often highly motivated to engage in caring activities, such as preparing food for the animal. Third, animals elicit positive affect and accordingly, patients showed significantly more positive and, respectively, less neutral emotions during AAT compared to conventional therapy sessions. We found considerable differences between patients, indicating that some of them did profit more than others. Future research needs to address this effect closer since it will be important for practice to identify characteristics of patients who might profit and others that don’t. Moreover, we did see statistically significant effects over time, but these were negligible compared to the effect of the intervention. Although total social behaviour decreased over time, the patients still showed much more social behaviour during AAT compared to conventional therapy sessions at the end of the study. This result contributes to the discussion about novelty effects in AAT as it indicates that a novelty effect may be present but it might be considered as rather small.

The patient’s and the therapist’s statements correlated, indicating a good validity of the patient’s self-assessment, although this ability is sometimes questioned in brain-injured patients. AAT helps addressing the common deficits in affective responsivity and emotional expression of patients with acquired brain injury^4,5^ and could help to prevent depressive symptoms. Notably, emotional arousal is correlated with learning and memory^27^. Our finding of increased positive emotions, higher motivation and satisfaction during AAT supports the potential of AAT in cognitive rehabilitation therapy and social skills training after brain injury^28^.

Our study has several important limitations. Patients, therapists and the study team could not be blinded concerning the condition. Also, the study team that coded the videos was not blinded. We implemented detailed coding ethograms to minimize this effect and interrater reliability was high. Also, different measures found similar effects indicating validity of the outcomes. Both TBI and non-TBI patients were included in the study, and the patients differed in their functional impairments. However, TBI and non-TBI patients did not differ regarding total or cognitive functional impairment at study start, and we designed the study as a within-subject trial. This provided control for within-patient effects such as the individual rehabilitation progress. Since the results reflect only 19 participants, these cannot be generalized without future replication. A further limitation is that the study investigated only immediate effects of AAT. The concurrent implementation of AAT and conventional therapy make it impossible to identify cumulative effects of AAT that could lead to long-term symptom reduction. Our goal was to address short-term effects in a first step in a setting that can be controlled as much as possible and to gain knowledge about possible mechanisms of AAT in patients with acquired brain injury. Further studies should now address longer-term effects of AAT on social communication and interaction, especially in regard to control for novelty factors and transferability of the beneficial effects of AAT on social behaviour in patient daily life. In a pilot study on a population of post stroke aphasia patients, no significant difference between the effect of a classical speech therapy and a dog-assisted speech therapy on the patient verbal communication skills was found^11^. It is therefore crucial to further investigate the effectiveness of AAT on functional skills and rehabilitation outcome. In our study, we had a broad range of settings and therapeutic activities depending on the included species. We hypothesize that our results are based on common factors due to the presence of an animal such as experience of a relationship with an animal, rather than an effect of species or the specific setting that different animals imply. Future research should further investigate species specific effects and mechanisms of AAT.

This field study was integrated in the everyday life of stationary neurorehabilitation, meaning confounding variables could only be controlled within certain limits. This restricts the internal validity of the study but at the same time strengthens external validity of the results. We present a randomized controlled study with many different therapists conducting the therapy sessions as usual, with no manual for integrating animals into therapy. Together with the relatively high number of observed therapy sessions, providing adequate statistical power, these are clear strengths of the study.

In conclusion, findings from the present study demonstrate that patients with acquired brain injury showed higher social engagement, motivation and satisfaction during AAT sessions in comparison to conventional therapy sessions. Moreover, these effects were seen during AAT with different animal species. The results are clinically highly relevant because social competence is a prerequisite for quality of life, social integration and functioning within the community^19,20^. In addition, emotional involvement of patients during therapy is essential to the rehabilitation process. Patients with acquired brain injury undergoing neurorehabilitation often suffer from a lack of motivation and initiative, which are core factors for therapeutic success and enhancing neuroplastic changes and functional outcomes^29^. Integration of animals into therapy might therefore be a promising approach to improve therapeutic effects on socioemotional functioning in patients with acquired brain injury during neurorehabilitation.

## Methods

### Participants

Adult (≥18 years) inpatients in stationary neurorehabilitation with an acquired brain injury from either traumatic (TBI) or nontraumatic cause (non-TBI) were invited to participate in the study. For inclusion in the study, patients had to meet the following criteria: (a) be medically stable, (b) be able to walk or to be transported to the therapy-animal facility, (c) be able to interact with an animal autonomously, (d) have no medical contraindications (e.g. phobias or allergies), and (e) exhibit no aggressive behaviour towards the animals. The head physician proposed inpatients for the study and the patients were then screened for inclusion criteria. All the experiments were performed in accordance with relevant guidelines and regulations. The human-related protocols were approved by the Human Ethics Committee for Northwest and Central Switzerland (EKNZ), and all patients or their legal guardians provided written informed consent. The animal-related protocols were approved by the Veterinary Office of the Canton Basel-Stadt, Switzerland. AAT was performed according to the IAHAIO-guidelines^30^. No therapy session had to be ended early, and no adverse incidents occurred. The patients were offered the possibility to continue with AAT after the end of the study. The study was registered at ClinicalTrials.gov (Identifier: NCT02599766, date 09/11/2015).

### Study design and procedures

The study had a randomised controlled, within-subject design with repeated measurement and was conducted at a clinic for neurorehabilitation and paraplegiology in Switzerland (REHAB Basel). Patients were randomly assigned by the principal investigator, using random numbers generated with Microsoft Excel, to either start with an AAT session or a conventional therapy session (control). Patients and therapists were not blinded because animals were either present or not. Coders were not blinded because the animals were visible in the videos.

The study program included two experimental and two control therapy sessions per week over a six-week period, with a total of 24 therapy sessions (*N* experimental = 12, *N* control = 12) per patient. Due to illness of patients or therapists, some sessions had to be cancelled and some data were lost due to technical problems. This resulted in a total of 441 analysed therapy sessions within this study consisting of 222 AAT and 219 conventional therapy sessions. The experimental condition consisted of speech, occupational, or physiotherapy sessions including an animal (referred to as AAT). The control condition consisted of conventional speech, occupational, or physiotherapy sessions (treatment as usual).

First, therapists and patients chose a suitable animal for the AAT sessions. The animals involved in the project were horses, donkeys, sheep, goats, miniature pigs, cats, chickens, rabbits and guinea pigs. All animals were housed in the therapy-animal facility at REHAB Basel, had experience in working with brain-injured patients and were kept and handled according to the IAHAIO-standards^30^.

Every session lasted approximately 30 minutes. After each therapy session, the patients and therapists filled out the questionnaires. AAT- and conventional therapy sessions were conducted concurrently and pairwise with similar comparable therapeutic activities. This was planned such that the conditions alternated and the matching sessions respectively took place within two successive weeks to control for improvements over time. Matched AAT and conventional therapy sessions were conducted by the same therapist and controlled for time of day and day of the week. The AAT sessions were held at the therapy-animal facility at REHAB Basel in the presence of an AAT specialist who assisted the therapist.

Although therapy sessions were matched within one patient for activities, goals and setting, there was a great amount of variability between patients depending on the involved animal. However, in the animal-assisted therapy sessions, the procedure always followed a scheme: First, the patient and the therapist greeted the animal and then the therapist explained the therapeutic activity that had to be related with the presence of the animal. Examples for therapeutic activities were as reported in a previous paper^31^: Cutting vegetables and feeding it to the present guinea pigs (AAT session) versus cutting vegetables to make a salad (conventional occupational therapy/physiotherapy/speech therapy); building a course and walking through it with, for example, a minipig (AAT), versus building a course and walking through managing a ball (conventional occupational therapy/physiotherapy); cleaning the rabbit’s cage in the presence of the animal (AAT) versus cleaning furniture (conventional occupational therapy/physiotherapy/speech therapy); walking with a sheep and the therapist (AAT) versus walking with the therapist (conventional physiotherapy); reading questions about the involved, present animal and filling in the answers (AAT) versus reading questions about an animal in general and filling in the answers (conventional speech therapy). In the previous paper, we also presented the number of sessions held with the different species involved in the study^31^.

#### Behaviour analysis

All therapy sessions (N = 429) were videotaped with a handheld camera (Sony HDR-CX240). The videos were analysed with a behavioural coding system software (Observer XT 12, Noldus). Analyses were done continuously, defining each second of the video with the different variables as present or not for state behaviour variables. The percentage of the duration of each state variable in relation to the observed time period of a therapy session was calculated. Count variables were coded only if they occurred, and the total occurrence within a therapy session was calculated. All videos were coded according to a strict ethogram defined by detailed descriptions of the behaviours with inclusion and exclusion examples. The coding scheme was developed for the purpose of this study, based on previously published behaviour coding systems for studies on AAT in patients with dementia or autism spectrum disorder^15,32^. We modified our system only slightly according to the purpose of the present study population and the study aims to ensure comparability. Our coding scheme includes the dimensions “social behaviour” (Supplementary Table S1), “emotion” (Supplementary Table S2), “attention”, and animal presence (Supplementary Table S3). The results for the dimension “attention” were previously published^31^. Inter-rater reliability was measured by Cohen’s kappa. Before coding the actual data, each rater had to achieve an inter-rater reliability of *k* > 0.80. During the actual coding process, two follow-up assessments of agreement were conducted. No renewed training was necessary. Inter-rater reliability ranged between 0.81 and 0.95, which indicated excellent agreement among coders.

#### Outcomes

The primary outcome was patient total social behaviour, measured as the observed relative duration of verbal and non-verbal social communication and interaction of the patients via behaviour analysis (Supplementary Table 1). Verbal communication was defined as state behaviour and coded as active, reactive or undefined. Active verbal communication was initiated by the patient and was addressed to either the therapist or the animal, while reactive verbal communication was defined as direct answer to a question or as verbal reaction to a cue from the therapist. Non-verbal social communication and interaction was defined as state behaviour and included gaze (eye contact), body movement towards an interaction partner, and active physical contact. All variables could be coded in parallel and were defined as either towards animal or towards therapist. The patient’s displayed emotions were defined as state variable and comprised the mutually exclusive variables positive emotion, negative emotion, and neutral state. All behavioural categories or subcategories represent the percentage of the total duration of the respective behaviour in one therapy session.

The subcategories of measured social behaviour as well as mood, treatment motivation and satisfaction were defined as secondary outcomes. The multidimensional mood questionnaire (MDBF)^33^ was used to gather information about the patient’s mood during therapy sessions. Patients filled out the MDBF at the end of each session. We analysed the bipolar mood dimension (good-bad) ranging from 4 (*not at all good mood*) to 20 (*very good mood*). The patient’s treatment motivation was assessed by self-report and by the therapist using a visual analogue scale (VAS) where a cross could be made on a line ranging from 0 mm (*unmotivated*) to 160 mm (*motivated*). Satisfaction during the therapy sessions was assessed by the patients themselves and by the therapists using a VAS ranging from 0 mm (*unsatisfied*) to 160 mm (*satisfied*).

#### Statistical analysis

We estimated mean and standard deviation of the primary outcome on the basis of published literature regarding percentage of speaking time (M = 65%, SD = 20%-points)^34^ and defined an intervention effect between 5% and 10% as practically relevant. The simulation revealed a total of 19 participants (observed at 24 time points) to detect an average effect of 7.5% with a power of 80% at a significance level of 95%. We increased the final sample size to 22 to account for possible dropouts.

We used linear mixed models (LMM) to examine the effects of AAT sessions on the duration of displayed behaviours in patients with acquired brain injury, as compared to conventional therapy sessions. These account for the hierarchical structure of the data, i.e. 24 repeated measurements per patient. The model included the variable “condition” (AAT versus conventional therapy sessions) as fixed factor and a random intercept for “subject”. As effect size we used the coefficient (b) estimating the difference in percentages. Coefficients together with the 95% confidence intervals, *p*-values and F statistics were summarized in Table 1.

**Table 1:** Behavioural outcomes (in percentage of observed time during a therapy session).

Behaviour	Control M% (SD)	AAT M% (SD)	b	95% CI	df	F-value	*p*-value	ICC
Total social behaviour	8.68 (8.85)	29.22 (15.66)	20.47	18.32–22.62	1;421.00	351.51	<0.001*	0.21
Total verbal communication	19.04 (20.24)	25.40 (19.44)	6.32	3.77–8.86	1;420.91	23.76	<0.001*	0.57
Verbal active	6.31 (8.80)	13.65 (11.21)	7.30	5.87–8.73	1;421.12	100.76	<0.001*	0.44
Verbal reactive	13.51 (13.52)	11.02 (11.20)	−2.44	−3.91–−0.98	1;407.86	10.81	0.001*	0.67
Total non-verbal interaction^a^	5.26 (7.09)	31.94 (17.54)	26.65	24.29–29.01	1;421.53	492.65	<0.001*	0.12
Gaze	10.40 (12.76)	52.89 (21.22)	42.42	39.26–45.58	1;421.95	694.51	<0.001*	0.08
Body movement	4.33 (10.24)	28.37 (19.98)	24.06	21.36–26.77	1;421.40	306.35	<0.001*	0.18
Physical contact	1.53 (9.70)	18.32 (18.50)	16.84	14.19–19.49	1;421.16	156.10	<0.001*	0.09
Positive emotion	3.75 (4.57)	6.95 (7.47)	3.24	2.28–4.19	1;409.10	44.44	<0.001*	0.36
Negative emotion	0.59 (2.05)	0.31 (1.34)	−0.27	−0.58–0.04	1;409.14	2.85	0.09	0.09
Neutral emotion	94.20 (6.60)	92.73 (7.64)	−2.97	−3.97–−1.97	1;409.10	33.91	<0.001*	0.34

AAT: animal-assisted therapy, M: mean in % of observed time (^a^adjusted for possible parallel behaviour), SD: standard deviation, d: coefficient indicating difference in percentages (effect size), CI: confidence interval, df: degrees of freedom (numerator; denominator), ICC: Intraclass correlation coefficient, *statistically significant.

For all behaviours, the denominator “therapy on-going” was used. This ensured that the reference time (100%) only counted when the therapy was in process. The cumulative variables for “total social behaviour” and “non-verbal communication” were adjusted for possible parallel behaviour and behaviour that could only occur in the presence of an animal so that they could maximally add up to 100% during a therapy session. The intraclass correlation coefficient (ICC) was used to quantify between-patient effects. In a second step, we investigated time effects to account for possible improvement of the outcomes over time. For that, we additionally included time (time point 1–24) as fixed factor in the model. If time had a significant effect, we looked at time effects for both conditions separately and included both “AAT over time” and “control over time” as fixed effects in the model. Analysis of the questionnaires were also analysed via LMM with the same specifications as for the first model. We did not adjust for multiple comparisons regarding secondary outcomes since we had an exploratory aim with these.

All variables were visually checked for normality (histogram and Q-Q-plot). Model diagnostics of LMM included visual checks for normality and homogeneity of residuals. All data were approximately normally distributed. No data were excluded. Statistical analyses were performed with SPSS, Version 23 (IBM SPSS^®^ Statistics) and the significance level was set at *p* ≤ 0.05.

## Supplementary information

Supplementary Materials

## Data Availability

The datasets generated during and analysed during the current study are available from the corresponding author on reasonable request.
