# Supplementary material for: Effects of animal-assisted therapy on social behaviour in patients with acquired brain injury: a randomised controlled trial
Source: Sci Rep. 2019 Apr 9;9:5831. doi: 10.1038/s41598-019-42280-0 (PMC6456498; doi:10.1038/s41598-019-42280-0)
Supplement: Supplementary file 1 — Supplementary Materials [file 41598_2019_42280_MOESM1_ESM.docx]

**Effects of animal-assisted therapy on social behaviour in patients with acquired brain injury: a randomised controlled trial**

Karin Hediger, Stefan Thommen, Cora Wagner, Jens Gaab, Margret Hund-Georgiadis

**Supplementary Materials**

**Supplementary Table S1.** Ethogram for the dimension of social behaviour

| **Category** | | | **Definition** |
| --- | --- | --- | --- |
| Verbal | To therapist | Active | Self-initiated vocalizations/speech to therapist |
|  |  | Reactive | Vocalizations/speech as answer to a question, statement or prompt of a therapist |
|  | To animal | Active | Self-initiated vocalizations/speech to animal |
|  | Undefined | | Vocalizations that are not directed to a receiver, self-referred murmuring |
| Non-verbal | Gaze | To therapist | Eye contact with therapist, gaze to therapist, gaze is focused (and following) |
|  |  | To animal | Eye contact with animal, gaze to animal, gaze is focused (and following) |
|  | Body movement | To therapist | Body and/or head is turned in the direction of the therapist, the arms and/or the torso is being moved in the direction of the therapist, walking/going in the direction of the therapist |
|  |  | To animal | Body and/or head is turned in the direction of the animal, the arms and/or the torso is being moved in the direction of the animal, walking/going in the direction of the animal |
|  | Touch/physical contact | To therapist | Patient touches the therapist actively (therapist touching the patient is not included) |
|  |  | To animal | Touching, stroking, cuddling, brushing, feeding treats |

**Supplementary Table S2.** Ethogram for the dimension of emotion

| **Category** | | **Facial expression** | | **Posture** | **Vocalization** |
| --- | --- | --- | --- | --- | --- |
| Positive emotion | Happiness | Smiling, raised outer corners of the mouth, pushed-up cheeks, narrowed eyes, crow's feet wrinkles | | Touching others friendly | Laughter, direct verbalization of pleasure or joy |
| Neutral | Neutral | Neutral face | | No emotional posture | No emotional vocalization |
| Negative emotion | Anger | Lowered eyebrows, tense lower eyelids, compressed lips | | Physical aggression, fist shaking, gesture of distance | Angry voice, shouting, grumbling, swearing, direct verbalization of anger |
|  | Fear | Eyebrows raised and pulled together, raised upper eyelids, tensed lower eyelids, lips tightly stretched horizontally |  | Cringing, repetitive or agitated movements, wringing of hands, shaking | Screaming, shouting repetitively, audibly faster breathing, direct verbalization of fear |
|  | Sadness | Inner corner of eyebrows raised, mouth corners down, wrinkled forehead | | Head down, eyes down, head rested in hand | Weeping, crying, moaning, whining, direct verbalization of sadness |

**Supplementary Table S3.** Additional coded variables

| **Category** | | **Definition** |
| --- | --- | --- |
| Animal presence | Present | At least one animal is present in the room and noticed by the patient |
|  | Not present | No animal is present in the room |
| Therapy session | Ongoing | Ongoing therapy session and patient is visible |
|  | Not ongoing | Therapy session is interrupted, patient is not visible, patient leaves the room |
